# Cooperative interaction of MUC1 with the HGF/c-Met pathway during hepatocarcinogenesis

**DOI:** 10.1186/1476-4598-11-64

**Published:** 2012-09-11

**Authors:** Giray Bozkaya, Peyda Korhan, Murat Çokaklı, Esra Erdal, Özgül Sağol, Sedat Karademir, Christopher Korch, Neşe Atabey

**Affiliations:** 1 Department of Medical Biology and Genetics, Dokuz Eylul University, Medical School, 35340, Balcova-Izmir, Turkey; 2 Department of Pathology, Dokuz Eylul University, Medical School, 35340, Balcova-Izmir, Turkey; 3 Department of Surgery, Dokuz Eylul University, Medical School, 35340, Balcova-Izmir, Turkey; 4 University of Colorado Cancer Center-DNA Sequencing & Analysis Core, 12801 E. 17th Ave, Aurora, CO, 80045, USA

**Keywords:** Hepatocellular carcinoma (HCC), Hepatocyte Growth Factor (HGF)/c-Met, Mucin 1 (MUC1), Differentiation, Invasion

## Abstract

**Background:**

Hepatocyte growth factor (HGF) induced c-Met activation is known as the main stimulus for hepatocyte proliferation and is essential for liver development and regeneration. Activation of HGF/c-Met signaling has been correlated with aggressive phenotype and poor prognosis in hepatocellular carcinoma (HCC). MUC1 is a transmembrane mucin, whose over-expression is reported in most cancers. Many of the oncogenic effects of MUC1 are believed to occur through the interaction of MUC1 with signaling molecules. To clarify the role of MUC1 in HGF/c-Met signaling, we determined whether MUC1 and c-Met interact cooperatively and what their role(s) is in hepatocarcinogenesis.

**Results:**

MUC1 and c-Met over-expression levels were determined in highly motile and invasive, mesenchymal-like HCC cell lines, and in serial sections of cirrhotic and HCC tissues, and these levels were compared to those in normal liver tissues. Co-expression of both c-Met and MUC1 was found to be associated with the differentiation status of HCC. We further demonstrated an interaction between c-Met and MUC1 in HCC cells. HGF-induced c-Met phosphorylation decreased this interaction, and down-regulated MUC1 expression. Inhibition of c-Met activation restored HGF-mediated MUC1 down-regulation, and decreased the migratory and invasive abilities of HCC cells via inhibition of β-catenin activation and c-Myc expression. In contrast, siRNA silencing of MUC1 increased HGF-induced c-Met activation and HGF-induced cell motility and invasion.

**Conclusions:**

These findings indicate that the crosstalk between MUC1 and c-Met in HCC could provide an advantage for invasion to HCC cells through the β-catenin/c-Myc pathway. Thus, MUC1 and c-Met could serve as potential therapeutic targets in HCC.

## Background

Hepatocellular carcinoma (HCC) accounts for 85% to 90% of primary liver cancers and is the fifth most common cancer worldwide [1,2]. More than 250,000 deaths and 500,000 new cases occur globally each year [1,3]. One of the main reasons for the high mortality rate is the lack of effective treatments and the development of resistance to conventional chemotherapy and radiotherapy [4]. In recent years, improved knowledge of signaling pathways regulating HCC growth and progression has led to the identification of several novel molecular targets. One of the most promising signaling pathways for molecular therapy of HCC appears to be the Hepatocyte Growth Factor (HGF)/c-Met cascade [4-7].

HGF was first characterized as a factor that induces hepatocyte proliferation and as a motility factor of epithelial cells [8-11]. HGF acts on c-Met, a high affinity tyrosine kinase receptor, and mediates several cellular behaviors including cell survival, proliferation, migration morphogenesis, and angiogenesis [7-21]. Both c-Met and HGF are overexpressed during liver development and it is known that the signal elicited through binding of HGF to c-Met is one of the main stimuli for the G1-S progression in hepatocytes [13]. In mice, deficiency of either HGF or c-Met expression causes embryonic lethality and reduced liver size; whereas, liver-specific deletion of c-Met induces hepatocyte necrosis and steatosis [14,15]. Furthermore, HGF/c-Met signaling is also essential for liver regeneration. A severe impairment of liver regeneration in the conditional c-Met mutant mice has been reported [14].

In addition to regulating normal cellular function, c-Met is implicated in tumorigenesis. Aberrant c-Met signaling, c-Met mutations, c-Met amplification/overexpression, and autonomous growth control through autocrine signaling loops have been found to be associated with carcinogenesis [7-21]. Perturbation of HGF/c-Met signaling is also involved in aggressive liver tumors and causes poor prognosis in HCC [15,17]. Recently, You et al. [15] reported that c-Met represents a potential target of personalized treatment for HCC with an active HGF/c-Met pathway. While overexpression of c-Met has been found to be associated with decreased 5-year survival in patients with HCC [17], deficiency of c-Met in hepatocytes has been reported to initiate tumorigenesis in liver [18]. Stoelting et al. [18] published that a defect in a c-Met-mediated signaling increases chemically-induced tumor initiation in liver. It also has been reported that the c-Met regulated gene expression signature characterizes a subset of HCC with aggressive phenotypic behavior and poor prognosis [20]. Although inappropriate HGF/c-Met signaling is involved in all of these biological processes, *in vivo* responses are rarely controlled by one signal; rather, interactions of multiple signaling pathways are involved. Recent studies have demonstrated additional roles for the HGF/c-Met signaling cascade in cancer through cross-talk with other signaling cascades, including integrins, class B plexins, proteoglycan CD44, G-protein coupled receptors, and many other receptor tyrosine kinases [21]. Many of these combinatorial signal interactions lead to augmentation of HGF/c-Met signaling and also contribute to therapeutic resistance. Recently, it has been reported that c-Met interacts with Mucin 1 (MUC1) and catalyzes the phosphorylation of the MUC1 cytoplasmic C-terminus in pancreatic cancer cells [22]. MUC1 is the best-characterized membrane-bound mucin that is expressed in most epithelial cells and is aberrantly overexpressed in various cancers, including breast, ovarian, lung, colon, and pancreatic carcinomas [23,24]. Although MUC1 expression correlates with high grade, metastasis potential, and poorer survival rate in breast cancer [25], the studies about MUC1 expression level in HCC are controversial. In some studies elevated MUC1 levels have been reported, while in other reports no differences have been found [26,27]. It also has been published that the oncogenic effects of MUC1 are dependent on the cellular context [28]. Furthermore, it is believed that different biological responses produced by MUC1 arise due to the particular repertoire of signaling molecules that interact with MUC1 [29].

In this study, we hypothesized that the HGF/c-Met signaling pathway might play diverse roles in hepatocarcinogenesis, depending on the MUC1 status of the cells. To test this hypothesis, we first analyzed MUC1 and c-Met expression levels in HCC cell lines. In our previous studies, we characterized the differentiation status of HCC cell lines as “well-differentiated” and “poorly-differentiated”. Poorly-differentiated, highly motile and invasive HCC cell lines that display a mesenchymal phenotype were usually deficient in the expression of hepatocyte lineage markers. However, well-differentiated cell lines, which have limited motility and invasion ability and which display an epithelial phenotype, shared many feature with hepatocytes [30,31]. In this study we observed that poorly-differentiated HCC cell lines overexpressed both MUC1 and c-Met, whereas well-differentiated ones expressed little or no amount of the MUC1 and c-Met proteins. To support these data we also analyzed MUC1 and c-Met expression patterns in primary HCC tissues, as well as in normal and cirrhotic liver samples. We found that both c-Met and MUC1 expression were increased during hepatocarcinogenesis and correlated with the differentiation status of HCC tissues. When we tested the hypothesis that MUC1 might form a complex with c-Met in the HCC cells, we observed an interaction between MUC1 and c-Met that was down-regulated under HGF stimulation. We then demonstrated that activation and inhibition of HGF/c-Met signaling and silencing of MUC1 altered the activation of the c-Met target genes, and cellular motility and invasion.

## Results

### Both MUC1 and c-Met are overexpressed in poorly-differentiated HCC cell lines

**Figure 1:**
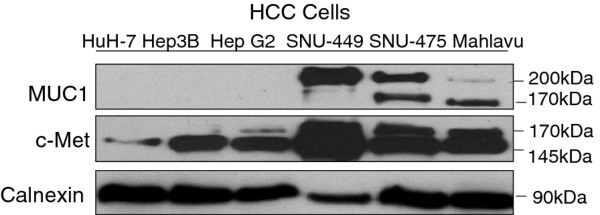
**Expression analysis of MUC1 and c-Met in HCC cell lines.** Total cell lysates were extracted from HCC cell lines to detect MUC1 and c-Met protein expression by immunoblotting assay. Two bands were detected for MUC1 due to posttranslational modification. Precursor protein band (170 kDa) and biologically active transmembrane β-subunit (140 kDa) of c-Met were detected. Calnexin was used to verify equal protein loading and transfer.

When we analyzed the expression of MUC1 and c-Met receptor tyrosine kinase in HCC cell lines (authenticated by DNA profiling), the cell lines fell into two groups based on the two expression profiles. Both MUC1 and c-Met were expressed highly in SNU-475, SNU-449, and Mahlavu cell lines, which had previously been characterized as poorly-differentiated HCC cells. In contrast, both MUC1 and c-Met were poorly or not expressed in HuH-7, Hep3B, and Hep G2 cells, which had previously been defined as well-differentiated HCC cells [30-32]. We detected two MUC1 bands, one of which represents the heavily glycosylated mature form (<200 kDa) and the other represents the poorly glycosylated form (~170 kDa) of MUC1 [33]. Similar to the MUC1 protein levels, expression of both the 170-kDa precursor c-Met and the 145-kDa β subunit of c-Met were higher in poorly-differentiated cell lines than in the well-differentiated cells (Figure 1). 

### The levels of both MUC1 and c-Met expression are higher in HCC tissues than in normal and cirrhotic liver samples

**Figure 2:**
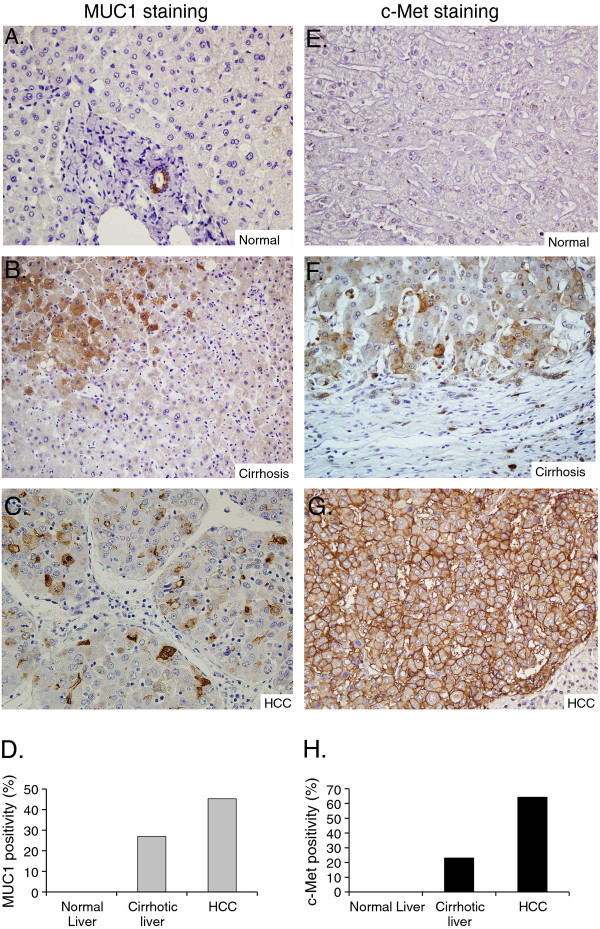
**Analysis of MUC1 and c-Met expression in normal and cirrhotic liver tissue, and HCC.** Negative MUC1 expression in normal hepatocytes and positive MUC1 expression was localized to bile ducts (×400) (**A**). Cirrhotic liver tissue showed weak, diffuse cytoplasmic MUC1 staining (×400) (**B**). HCC displayed intense MUC1 staining (×400) (**C**). Each column represents histologically classified liver tissues (normal liver, cirrhotic liver, HCC) with the height representing the ratio of positive staining for MUC1 (**D**). c-Met expression in normal liver tissue showed very weak or no immunoreactivity (×400) (**E**). Cytoplasmic c-Met staining in hepatocytes in the cirrhotic liver tissue (×400) (**F**). HCC displayed positive cytoplasmic and strong membranous c-Met staining (×400) (**G**). Comparison of the ratios of positive staining for c-Met in normal liver, cirrhotic liver, and HCC tissues (**H**).

MUC1 and c-Met expression profiles were analyzed by using immunoperoxidase staining. Expression of c-Met and MUC1 were examined in normal liver (n = 18), cirrhotic liver (n = 26), and HCC (n = 42) tissue samples. The clinicopathological characteristics of HCC patients are shown in Table 1. In normal liver tissues, both MUC1 (Figure 2A) and c-Met (Figure 2E) staining were observed in interlobular bile ducts, but not in normal hepatocytes. On the other hand, positive staining for MUC1 (Figure 2B) and c-Met (Figure 2F) were observed in 27% and 23% of cirrhotic liver tissues sections, respectively. In cirrhotic livers, c-Met was expressed in proliferating cholangiol and lymphocytes in fibrous septa as well. MUC1 staining was mainly cytoplasmic in tumor cells. However, some canalicular staining pattern was also observed, especially in areas where tumor cells had formed pseudoglandular structures, which are not readily identified as bile duct differentiation morphologically on Hematoxylen and Eosin (H&E sections). In HCC tissues, we observed 45% (19/42) positive staining for MUC1 (Figure 2C) and 64% (27/42) positive staining for c-Met (Figure 2G). The staining was membranous (4%) or cytoplasmic (4%) or cytoplasmic and membranous (92%) in reactive tumor cells for c-Met. In c-Met positive tumor samples, 11% were scored as one positive, 59% were scored as two positive and 30% were scored as three positive. Among MUC1 positive HCC patients 32% were scored as one positive, 53% were two positive and 16% were scored as three positive.

**Table 1:** Clinicopathological characteristics and MUC1 and c-met immunostaining of tumors from primary HCC patients

	**Parameters**	**n (%)**
Gender	Male	38 (91)
	Female	4 (9)
Tumor Size (cm)	≤3	22 (52)
	>3	20 (48)
Nodule number (n)	≤5	37 (88)
	>5	5 (12)
Etiology	Viral	39 (93)
	Alcohol	3 (7)
Tumor Grade	Well	7 (17)
	Moderate	29 (69)
	Poor	6 (14)

**Figure 3:**
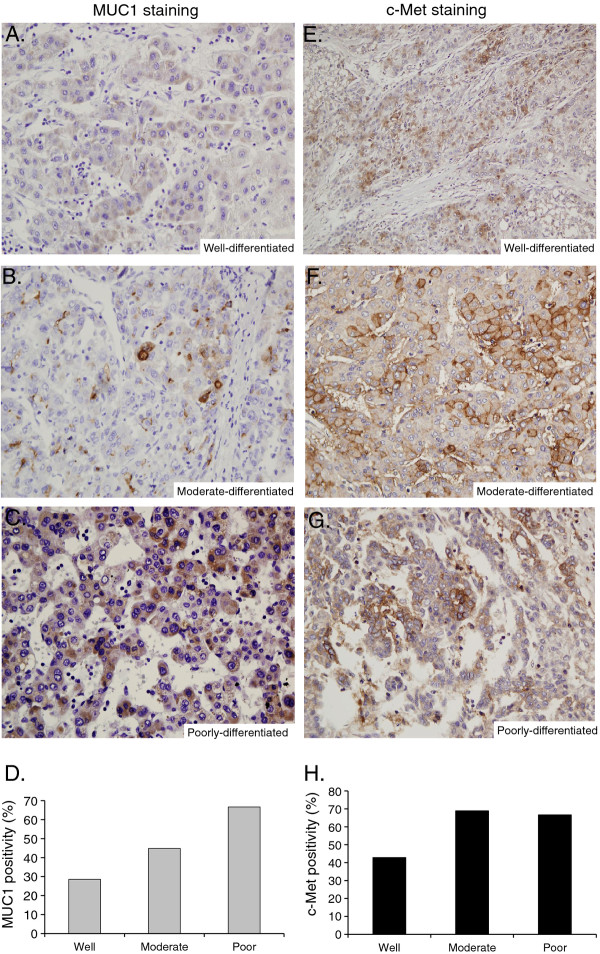
**Immunohistochemical characterization of MUC1 and c-Met expression in HCC tissues relative to tumor differentiation status.** Tissue sections from tumors with well (**A, E**), moderate (**B, F**), and poor (**C, G**) differentiation were assayed for MUC1 (**A,B,C**) and c-Met (**E, F, G**) expression by immunohistochemistry. Each column represents the ratio of positive staining for MUC1 (**D**) and c-Met (**H**) in well-, moderate-, and poorly-differentiated HCC. (E: 200X; A-D, F,G: 400X magnification).

The levels of both MUC1 and c-Met expression were significantly higher in cirrhotic samples than in normal tissues (p < 0.05 and p = 0.0025, respectively). The expression levels of MUC1 and c-Met in HCC were statistically greater in comparison to those observed in both normal (p = 0.0008 and p = 0.0001, respectively) and cirrhotic liver tissues (p = 0.005 and p < 0.0001, respectively) (Figure 2 D-H). There was no correlation between staining patterns or intensity and none of the clinicopathological data (p > 0.05). Since both MUC1 and c-Met expression levels were correlated with the differentiation status of cell lines, we investigated whether this profile was observed in patient samples. MUC1-positive HCC tissues were grouped as follows: 2/7 (29%) well-, 13/29 (45%) moderately-, and 4/6 (67%) poorly-differentiated (Figure 3A-D). The differences between the groups were not statistically significant. The levels of c-Met positive staining were 43% in well-, 69% in moderately- and 67% in poorly-differentiated HCC tissues. No significant differences were found between the groups (p > 0.05) (Figure 3E-H).

### MUC1 interacts with c-Met in Mahlavu and SNU-449 HCC cells

**Figure 4:**
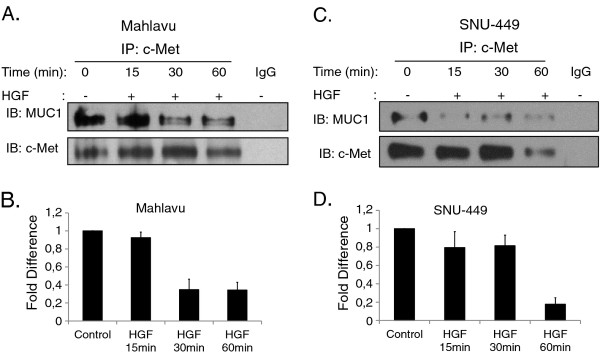
**Identification of MUC1 as an interaction partner for c-Met and effect of HGF on this complex**. Endogenous MUC1/c-Met interaction was carried out in a total cell lysate from Mahlavu cells (**A**) and SNU-449 (**C**) using anti-c-Met antibody by IP. The immunoblotting (IB) with MUC1 showed co-precipitation of endogenous c-Met and MUC1 in unstimulated and HGF stimulated cells. Anti-c-Met antibody was probed to the membrane as a loading control. There was no detectable c-Met and MUC1 in immunoprecipitates prepared with IgG as an IP-control. MUC1 signal intensities were measured by scanning the ECL exposed films with a densitometer. The relative MUC1 intensities (mean ± S.E.) for the different treatments relative to the levels present in the untreated control sample are compared in the bar graphs. Graphs represent data obtained from Mahlavu cells (**B**) and SNU-449 cells (**D**).

To determine whether c-Met interacts with MUC1, we performed co-immunoprecipitation by using anti-c-Met antibody in the Mahlavu and SNU-449 cells. As presented in Figure 1 Mahlavu cells express poorly-glycosylated form of MUC1 and SNU-449 express heavily-glycosylated form of MUC1. IP results showed that c-Met co-precipitates with MUC1 both in Mahlavu cells (Figure 4 A, B) and SNU-449 cells (Figure 4 C, D). These results indicated that both heavily and poorly glycosylated forms of MUC1 can bind the c-Met receptor in the HCC cell lines that we tested. However, we observed that when compared to Mahlavu cells, a lower amount of MUC1 is bound to c-Met in SNU-449 cells which express the heavily glycosylated form of MUC1. As a control, there was no detectable MUC1 and c-Met protein in the immunoprecipitates prepared with IgG alone.

### MUC1/c-Met interaction and MUC1 level are down-regulated under HGF stimulation

**Figure 5:**
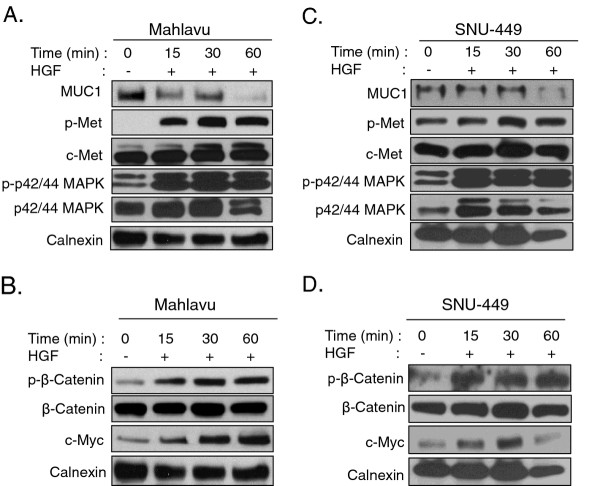
**Activation of c-Met signaling pathway by HGF administration induced p42/44-MAPK and β-catenin phosphorylation.** Overnight starved Mahlavu (**A, B**) and SNU-449 (**C, D**) cells were stimulated with medium alone and with HGF for 15, 30, and 60 min. Total cell lysates were then analyzed with Western blotting. Blots were probed with anti-p-Met, anti-c-Met, anti-MUC1, p-p42/44-MAPK, p42/44-MAPK, anti-p-β-catenin, anti-β-catenin, anti-c-Myc, and anti-calnexin antibodies.

To assess whether activation of c-Met affects its interaction with MUC1, Mahlavu and SNU-449 cells were treated with HGF (40 ng/ml) for 15, 30, and 60 min. HGF stimulation caused a decrease in the amount of MUC1 that co-immunoprecipitated with c-Met in a time-dependent manner both in Mahlavu (Figure 4 A, B) and SNU-449 cells (Figure 4 C, D). To further assess the effects of HGF-induced c-Met activation on the MUC1 expression, we prepared whole cell lysates from Mahlavu and SNU-449 cells after 15, 30, and 60 min of HGF treatment. Upon HGF binding, a gradual increase was observed in the tyrosine phosphorylation of the catalytic kinase domain at the autophosphorylation site [pY1234/1235] of c-Met, which reached a plateau at 30 min of HGF stimulation (Figure 5 A, C). Intriguingly, synchronous with the c-Met activation, a marked decrease was observed in MUC1 protein level. To test the hypothesis that the loss of MUC1 is due to cleavage of ECD or loss of the protein including the intracellular domain, we analyzed MUC1 levels in conditioned media and in cytosolic extracts of MUC1 positive HCC cells in the presence or absence of HGF. While MUC1 bands were detected in all cell lysates as expected, we did not observe any MUC1 band in conditioned media (Additional file 1 Figure S1A). We then used a second method for quantitative determination of MUC1 antigen in conditioned media. Similarly, no MUC1 was detected in the conditioned media from HCC cells by immunoassay analysis. In addition, we analyzed the MUC1 protein level in cytosolic extracts in parallel with total cell extracts. We obtained a similar pattern for MUC1, which decreased by HGF induction in a time-dependent manner both in cytosolic (Additional file 1 Figure S1B) and in total cell extracts. If loss of MUC1 was due to cleavage of ECD, then we should have observed MUC1 bands of lower MW (approximately 30 kDa lower) in cytosolic extracts. As we did not observe smaller MUC1 bands, we could argue that the loss of MUC1 is not due to the cleavage of ECD of MUC1 in HCC cell lines.

### HGF treatment increased phosphorylation of c-Met, and β-catenin and expression of c-Myc

To elucidate the role of MUC1/c-Met interaction and its down-regulation by HGF, we investigated signaling proteins downstream of both c-Met and MUC1. It is known that β-catenin interacts with both c-Met and MUC1 [34-37]and activation of c-Met effects β-catenin expression and phosphorylation at Ser552 (p-β-catenin-Ser552) resulting in its nuclear-localization [37,38]. Although expression of β-catenin was not affected significantly at the protein level by HGF-stimulation in HCC cells that we have tested, p-β-catenin levels gradually increased in response to HGF induction in a time-dependent fashion. Since phosphorylation at Ser552 plays a role in nuclear translocation of β-catenin, we further assessed expression of c-Myc, which is a β-catenin target gene [34,39]. Expression of c-Myc increased in parallel with β-catenin phosphorylation under the same conditions (Figure 5 B, D). We also determined that β -catenin interacts with MUC1; however, HGF treatment did not affect their interaction (data not shown).

### Inhibition of c-Met by SU11274 restores down-regulation of MUC1 by HGF

**Figure 6:**
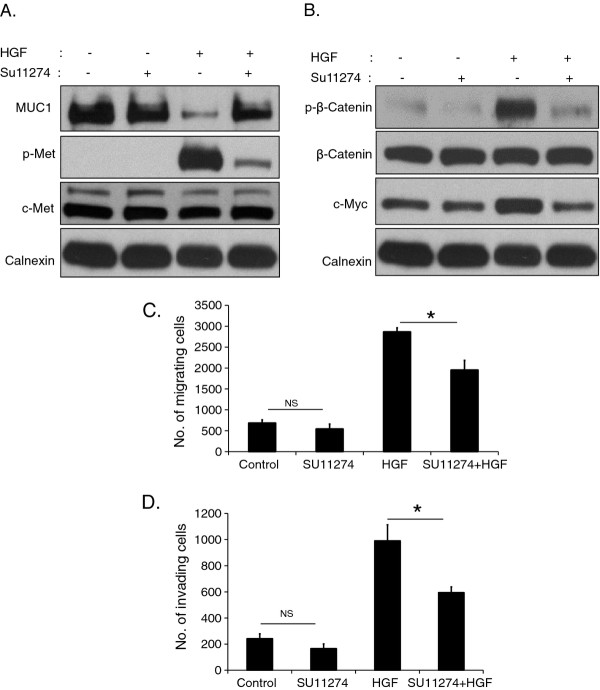
**SU11274 selectively inhibited c-Met phosphorylation and downstream signaling.** Mahlavu cells were incubated with medium alone or with SU11274 overnight. Then cells were treated with or without HGF for 60 min. Total protein lysates were analyzed by immunoblotting. Membranes were blotted with anti-p-Met, anti-c-Met, anti-MUC1 (**A**), anti-p-β-catenin, anti-β-catenin, anti-c-Myc (**B**), and anti-calnexin antibodies. SU11274 pretreated cells and control cells were seeded in the upper chamber of Boyden chambers. No HGF or 40 ng/ml HGF was added to the lower chamber. Following 24 h incubation, cells that had migrated or invaded onto the lower surface were stained and counted. Bars represent mean number ± S.E. of migrating (**C**) or invading (**D**) cells (* indicates p < 0.05, NS: not significant).

We showed above that activation of c-Met led to decreased MUC1 protein expression in a time-dependent manner. To further define the influence of c-Met activation on MUC1 down-regulation, we used SU11274, a specific inhibitor of c-Met, to block c-Met activation [40]. HGF induced phosphorylation of c-Met at the activation loop site phosphoepitope [pY1234/1235] was reduced by more than half compared to the HGF stimulated condition by SU11274 (Figure 6A, p -Met panel). Using SU11274, we restored HGF induced down-regulation of MUC1 and demonstrated that MUC1 down-regulation was correlated with c-Met activation in our experimental system (Figure 6A). Moreover in the presence of HGF, SU11274 also inhibited HGF-induced β-catenin phosphorylation and c-Myc expression (Figure 6B). Similar results were observed in SNU-449 cells (data not shown). 

### Inhibition of c-met decreases cell motility and invasion but does not alter proliferation of Mahlavu cells

To further evaluate the role of MUC1 downregulation by HGF treatment, we determined whether disruption of this complex affects biological behavior of HCC cells by performing adhesion, proliferation, migration, and invasion assays. For this aim, we analyzed migration and invasion of cells by using a modified Boyden’s chamber and matrigel invasion chambers, respectively. We first showed that HGF alone significantly enhanced the migratory and invasive capacities of Mahlavu cells (4- and 5-fold, respectively p < 0.0001). When phosphorylation of c-Met was inhibited by SU11274, there were no significant differences in the extent of cell migration and invasion under basal conditions (p > 0.05); however, in the presence of HGF, the addition of SU11274 caused sharp decreases in cell motility (p < 0.0001) and invasion (p < 0.001; Figure 6C, D). Although cellular motility and invasion were affected profoundly by activation and/or inhibition of c-Met signaling, adhesion and proliferation of cells did not changed significantly under similar experimental conditions (data not shown).

### Silencing of MUC1 improves HGF induced c-Met signaling

**Figure 7:**
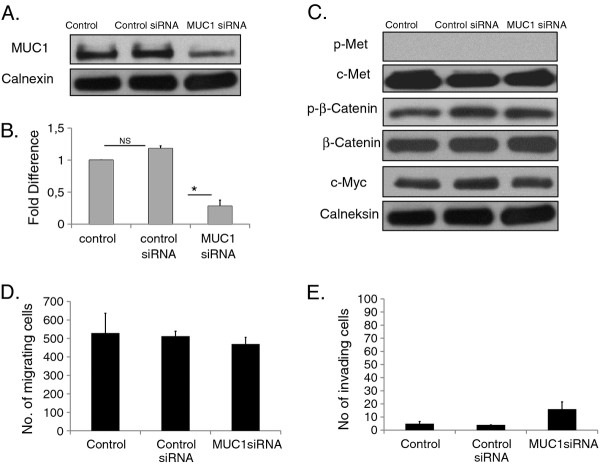
**Inhibition of MUC1 in Mahlavu cells.** Inhibition of MUC1 protein expression was demonstrated by siRNA gene silencing targeting of MUC1 mRNA. Mahlavu cells were transfected with MUC1-specific siRNA and control siRNA and analyzed using immunoblotting with anti-MUC1 antibody (**A**). Calnexin was blotted as internal loading control. Relative quantification of the MUC1 bands was done using ImageJ. Graph represents signals obtained from MUC1 bands. Error bars indicate SEM (*indicates p < 0.05, NS: not significant) (**B**). Total cell lysates from Mahlavu cells transfected with the control and MUC siRNAs and control siRNA were analyzed by immunoblotting. Membranes were blotted with anti-p-Met, anti-c-Met, anti-p-β-catenin, anti-β-catenin, anti-c-Myc and anti-calnexin antibodies (**C**). MUC1 knock-down cells and control cells were seeded in the upper chamber of Boyden chambers. After 24 h incubation, migrated and invaded cells were counted. Bars represent the mean ± S.E. of migrating (**D**) or invading (**E**) cell number (* indicates p < 0.05).

**Figure 8:**
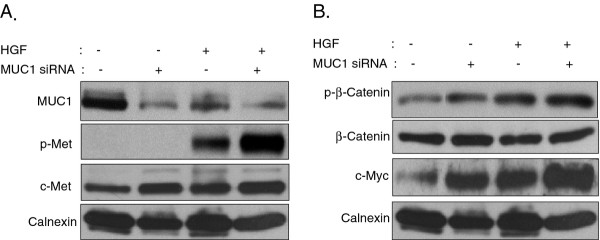
**Consequences of MUC1 silencing on downstream signaling molecules.** Mahlavu cells were transfected with siRNA targeted to MUC1 for 72 h followed by 60 min HGF stimulation. Controls included no treatment and MUC1 siRNA-treated cells. Total protein lysates were analyzed by immunoblotting. Membranes were blotted with anti-p-Met, anti-c-Met, anti-MUC1, anti-p-β-catenin, anti-β-catenin, anti-c-Myc, and anti-calnexin antibodies.

To determine whether MUC1 down-regulation is responsible for HGF induced c-Met and β-catenin phosphorylation in Mahlavu cells, cells were transfected with control or MUC1 siRNA oligos. siRNA silencing of MUC1 caused a significant (p < 0.05) down-regulation of the MUC1 protein level (Figure 7A, B). Control siRNA has no effect on MUC1 expression, nor on c-Met and β-catenin signaling (Figure 7C). Moreover, control siRNA did not have any effect on cellular motility (p > 0.05) (Figure 7D), invasion (p > 0.05) (Figure 7E) and proliferation of Mahlavu cells. In the absence of HGF, siRNA silencing of MUC1 has no effect on c-Met signaling, cell motility, and invasion of Mahlavu cells (Figure 7C, E). When Mahlavu cells, transfected with MUC1 siRNA for 72 h, were stimulated with HGF for 60 min, significant increases in the expression and phosphorylation of c-Met were observed compared to untreated cells (Figure 8A). Moreover, when MUC1 knockdown cells are treated with HGF for 60 min, the additive effects of both HGF induction and MUC1 silencing were seen in the p-β-catenin levels and its target protein, c-Myc (Figure 8B).

### Silencing of MUC1 increases the cell migration and invasion capability induced by HGF

**Figure 9:**
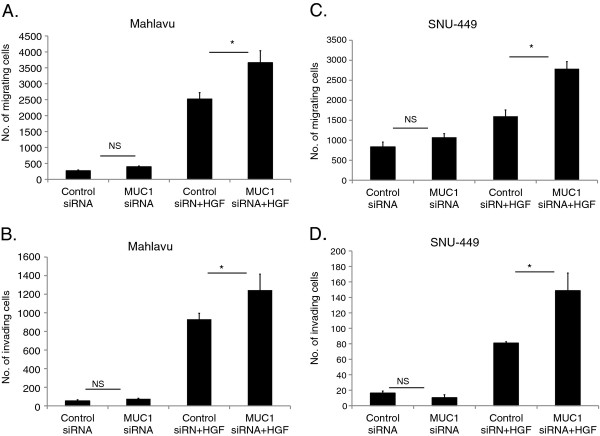
**Effect of MUC1 silencing on the cellular motility and invasion.** MUC1 and control siRNA treated cells were seeded in the upper chamber of Boyden chambers. Medium with or without HGF was added to the lower chamber. After 24 h incubation, migrated Mahlavu (**A**) and SNU-449 (**C**) cells and invaded Mahlavu (**B**) and SNU-449 (**D**) cells were counted. Bars represent the mean ± S.E. of migrating (**A,C**) or invading (**B,D**) cell number (* indicates p < 0.05).

MUC1 knockdown cells were significantly more motile and invasive compared to wild type cells under HGF treatment (p < 0.001) (Figure 9). However, there were no statistically significant differences in invasion and motility for MUC1 siRNA treated cells compared to the control siRNA treated cells without HGF stimulation (p > 0.05). Moreover, MUC1 silencing did not alter either the basal or the HGF-induced cell adhesion and proliferation capabilities of Mahlavu cells (data not shown).

## Discussion

The HGF/c-Met pathway is an important regulator of tumor invasion and metastasis in various types of human cancer. Activating mutations and overexpression of c-Met have been associated with intrahepatic metastasis, vascular invasion, poor prognosis, and drug resistance in HCC [19-21,41]. The role of c-Met in the phosphorylation of MUC1 in pancreatic cancer progression has been recently reported [22]. Remarkably, overexpression of MUC1 in cancer cells correlated with tumor metastasis, poor prognosis, and resistance to chemotherapeutic agents [23,42], which suggest that MUC1 and c-Met might work together. However, to our knowledge, there is no published study on the role of the potential crosstalk between MUC1 and c-Met in HCC. In this study, we investigated the possible roles of MUC1 and c-Met in hepatocarcinogenesis and how they interact in this process. We showed that expression patterns of c-Met and MUC1 correlated with each other and with the differentiation status of HCC cell lines and tumor tissues. Moreover, both c-Met and MUC1 expression levels were significantly higher in HCC tissues than in cirrhotic and normal liver samples. Furthermore, overexpression of c-Met and MUC1 was observed in poorly-differentiated and highly motile and invasive HCC cell lines. Correlated with these findings, we found MUC1 and c-Met to be expressed at higher levels in poorly-differentiated primary HCC tissues than in well- differentiated tumors. The overexpression of the MUC1 and c-Met proteins seen in cirrhotic liver and HCC tissues was not observed in normal liver biopsies. This suggests that the expression of MUC1 and c-Met increases during transformation of normal liver to HCC. While this paper was under preparation You et al. [15] demonstrated that c-Met positive HCC cell lines display a mesenchymal phenotype compared to the c-Met negative cells, which have an epithelial phenotype [15]. The results of You et al. [15] support our observations that show poorly-differentiated HCC cells over-express c-Met, whereas well-differentiated ones do not. Additionally, herein we demonstrate MUC1 over-expression in the poorly-differentiated mesenchymal-like HCC cells. Since c-Met is a cell differentiation marker, selective expression of MUC1 and c-Met in poorly-differentiated cell lines might contribute to the epithelial-mesenchymal transition during hepatocarcinogenesis.

In addition to their complementary role in HCC development and progression, in this study we demonstrate an association between MUC1 and c-Met in Mahlavu cells. Singh et al. [22] reported a finding supporting our observations; namely, they reported that c-Met is an interaction partner of MUC1 in pancreatic tumor cells. In addition to the MUC1/c-Met association, we also showed that HGF stimulation down-regulated the MUC1/c-Met interaction. When we treated HCC cells with HGF, we observed a remarkable reduction in MUC1 levels in a time-dependent manner, while c-Met expression was unaffected. Additionally, inhibition of c-Met activation restored MUC1 expression, which clearly demonstrated that HGF facilitated MUC1 down-regulation. Hence, we argue that in our experimental system the reduction in the MUC1/c-Met interaction is due to decreased MUC1 protein levels in response to HGF inductions. Contrarily, Singh et al. [22] demonstrated that under HGF stimulation up to 120 min, MUC1 promoted endocytosis and increased turnover of c-Met. To understand the basis of the differences between HCC and pancreatic cancer cells we performed a time course experiment. We did not observe a noticeable down-regulation of c-Met between MUC1 over-expressing and MUC1 negative HCC cells during 16 h of HGF induction (Additional file 2 Figure S2). This is supported by our IHC analysis which demonstrated that the elevated levels of both MUC1 and c-Met expression in HCC tissues were correlated positively with each other; namely, no detectable c-Met down-regulation was observed in tissues that over-expressed MUC1. Our results also showed that silencing of MUC1 by siRNA upregulated HGF-mediated c-Met signaling network. Overall, our work is distinctive in demonstrating for the first time the effect of HGF induction both on MUC1/c-Met association and MUC1 protein levels without altering the c-Met expression pattern. Moreover, in addition to the modulation of MUC1 by c-Met, we have demonstrated the regulation of c-Met activity by MUC1.

Singh et al. [22] showed that phosphorylation of the MUC1 cytoplasmic tail by c-Met activation enhances its interaction with p53, which leads to a reduction in the transcription of MMP1 and ultimately decreases invasion when MUC1 is overexpressed. In contrast, we have shown that MUC1 over-expressing HCC cell lines are highly motile and invasive. It has been reported that loss of p53 or the presence of abnormal forms of the p53 protein are common phenotypes in HCC cell lines including Mahlavu. Also, mutations of the p53 gene have been frequently detected in recurrent HCC patients [43-45]. The differences between the results of Singh et al. [22] and ours might be related to the p53 status of the HCC cells. To test this hypothesis, we evaluated functional p53 expressions in HCC cell lines examined in this study, and found no correlation between p53 status of HCC cells and MUC1 and/or c-Met co-expression or association (Additional file 3 Table S1). Therefore, the downstream signaling network of MUC1/c-Met association might be independent of p53 in HCC.

Previous studies have indicated that c-Met interacts with β-catenin at the inner side of the hepatocyte membrane in normal rat liver [34]. After HGF stimulation, c-Met associated with β-catenin dissociates and translocates to the nucleus [34,35]. Besides being associated with c-Met, β-catenin also interacts with the cytoplasmic tail of MUC1 [36]. Putting these findings together, we questioned whether MUC1 regulates c-Met activity via β-catenin. Since we did not observe any alteration in the MUC1/β-catenin interaction depended on HGF stimulation, we therefore examined the effect of HGF induction on β-catenin expression and phosphorylation. Our studies demonstrated that the phosphorylation of β-catenin at Ser-552, which mediates β-catenin migration to the nucleus [37], was increased by HGF stimulation in a time-dependent manner. The interaction of c-Met RTK and β-catenin was reported by Monga SPS et al. in primary rat hepatocytes and they also identified the domain in β-catenin which is responsible for this interaction in rat hepatoma cells [34,46]. Although we demonstrated β-catenin/MUC1 and MUC1/c-Met interaction in poorly-differentiated HCC cells, we did not observe an interaction between β-catenin and c-Met in either Mahlavu or SNU-449 HCC cell lines in the presence or absence of HGF. The reason could be the deficiency of E-cadherin in poorly-differentiated HCC cell lines which we and others have reported previously [30,31,47]. We believe that this is an important question that should be addressed in future investigations.

Nuclear localization of β-catenin is primarily associated with the induction of c-Myc expression [34,39]. It has been reported that a rise in the p-β-catenin level resulted in increased expression of c-Myc, which is a β-catenin target gene [34,39]. HGF-induced elevation of p-β-catenin and c-Myc levels observed in our study implies that β-catenin participates in HGF-mediated c-Met signaling in HCC cells. When HGF-induced c-Met activation was blocked by a c-Met specific inhibitor, β-catenin activation and concurrently c-Myc expression were suppressed. Interestingly, when MUC1 was silenced, HGF induced expression and activation of c-Met increased markedly compared to control cells. In accordance with c-Met activation, β-catenin activity and c-Myc expression increased in MUC1-silenced conditions. These results clearly demonstrate that MUC1 is a potential regulator of HGF/c-Met mediated β-catenin activation and of Myc expression in HCC cells. Aberrant activation of β-catenin signaling has been observed frequently in HCC [30,48,49]; whereas, mutational activation of β-catenin signaling is found only in about 20-30% of HCCs [49]. Our results suggest that MUC1/c-Met crosstalk is one of the important regulatory mechanisms involving aberrant activation of β-catenin signaling in HCC.

As we reported previously, poorly-differentiated HCC cells are highly motile and invasive under basal conditions; whereas, well-differentiated cells are not [30]. Since MUC1 and c-Met are over-expressed and physically interact in poorly-differentiated HCC cells, we tested the role of this association on the invasive behavior of these HCC cells. It has been reported that MUC1 over-expressing pancreatic tumor cells have higher c-Met signaling activity and a greater tendency to metastasize when low levels of HGF are present in the tumor microenvironment [22]. This is supported by the finding that the invasive ability of pancreatic tumor cells decreased under HGF stimulation [22]. In this situation, the disruption of MUC1/c-Met interaction by HGF should decrease the invasive ability of HCC cells. However, the activation of HGF/c-Met signaling noticeably increased motility and invasiveness, despite MUC1 down-regulation of Mahlavu and SNU449 cells. As expected, the inhibition of the c-Met signaling network diminished HGF-induced cell motility and invasion by HCC cells. Interestingly, the knockdown of MUC1 increases invasion of Mahlavu and SNU 449 cells in response to HGF stimulation. It seems that HGF/c-Met mediated MUC-1 down-regulation or MUC1 silencing increased β-catenin activation and c-Myc expression, and this might confer a selective advantage for HCC cell invasion. This is supported by a report showing that elevated c-Myc expression via β-catenin phosphorylation, in response to HGF stimulation in colorectal carcinoma cells, is associated with a more tumorigenic and metastatic phenotype [39]. Since poorly-differentiated HCC cells are more invasive than well-differentiated ones, we performed a few experiments regarding the role of MUC1 and c-Met cooperation on the differentiation of HCC cells (data not shown). Our data support that although c-Met and MUC1 co-expression is very important for cellular differentiation of HCC cells, presence of HGF in the microenvironment determines cellular fate. However, due to the limitations of IHC studies it is difficult to interpret the data in HCC tissues. Although we used sequential sections from one paraffin embedded tumor tissue obtained from each patient for MUC1 and cMet staining, we cannot conclude that over-expression of MUC1 and c-Met occurs in the same cells in HCC tissues. In addition as we described above, HGF levels in the microenvironment affect the behavior of MUC1 and c-Met positive cells. Further studies are needed to clarify the role of MUC1 and c-Met cooperativity in HCC cells, including HGF status of the microenvironment together with c-Met, β-catenin, and Myc activation status in HCC tissues.

Overall, our data suggests that MUC1 and c-Met are overexpressed in poorly-differentiated HCC cell lines and tissues. Under basal conditions, MUC1 and c-Met interact with each other. The activation of HGF/c-Met signaling targets MUC1 to reduce its protein level, and thus prevents the down-regulatory effects of MUC1 on HGF/c-Met signaling and in turn increases motility and invasiveness. In support of this model, the inhibition of HGF-induced c-Met activation restores MUC1 expression, which results in decreased cellular motility and invasiveness. Furthermore, the silencing of MUC1 increases HGF induced c-Met activation as well as the invasion of Mahlavu and 449 cells, showing that MUC1 down-regulation is an important regulator of c-Met activation in HCC.

## Conclusions

In conclusion, the results of this study demonstrate the modulation of MUC1 by c-Met activation and provide the first evidence of the regulatory effects of MUC1 on c-Met activity in HCC. We propose that MUC1 and c-Met have complementary roles during hepatocarcinogenesis and that their interaction is important for HGF-induced cellular invasion and metastasis in HCC.

## Methods

### Cell culture

Human hepatocellular carcinoma cell lines HuH-7, Hep3B, HepG2, SNU-449, SNU-475, and Mahlavu were cultured in DMEM supplemented with 10% FBS, 100U/ml penicillin, 0.1 mg/ml streptomycin, 2 mM L-glutamine and 1% MEM non-essential amino acids solution in a humidified 5% CO_2_ incubator at 37°C. HCC cell lines were kindly provided by Dr. Mehmet Öztürk (Bilkent University, Ankara, TR). Authentication of cell lines was done by DNA profiling at the University of Colorado Cancer Center (UCCC) DNA Sequencing & Analysis Shared Resource (CO, USA) using Applied Biosystem’s Identifiler kit (PN 4322288). Hepatocyte growth factor/scatter factor (HGF) was from R&D Systems (MN, USA). HGF (40 ng/ml) was used at specific time points after overnight starvation in DMEM with 2% FBS. For the inhibition of c-Met, SU11274 (Calbiochem 448101), was added to the cultures upon start of starvation. DMSO was used as solvent control of SU11274, which is dissolved in DMSO (Applichem).

### Immunoprecipitation

Total cell lysates for immunoprecipitation (IP) and immunoblotting (IB) were prepared from HuH-7, Hep3B, HepG2, SNU-449, SNU-475, and Mahlavu cells with modified RIPA buffer (50 mM Tris-Cl pH 7.4, 150 mM NaCl, 1 mM EDTA pH 8.0, 1% NP-40, 1x protease inhibitor cocktail (Roche, 11836153001) 1 mM NaF, 1 mM Na_3_VO_4_. Protein concentrations of samples were determined by the BCA assay following the manufacturer’s instructions (Pierce, IL, USA). 1000 μg of total lysate was used to analyze the interaction between c-Met and MUC1 in Mahlavu cells. Samples were incubated with 4 μg anti-MUC1 (sc-7313) or anti-c-Met (c-28) (sc-161) antibodies for 2 h at 4°C, and then Gamma-Bind Sepharose beads (Amersham 17-0886-01) were added to the mixture and further incubated overnight at 4°C. IP samples were then washed three times with IP washing buffer (50 mM Tris-Cl pH 7.4, 150 mM NaCl, 1 mM EDTA pH 8.0, 1% NP-40, 0.1x protease inhibitor cocktail (Roche) 0.1 mM NaF, 0.1 mM Na_3_VO_4_). Samples were re-suspended in 2x loading dye, boiled for 5 min at 95°C, and bound proteins were analyzed by immunoblotting as described below.

### Immunoassays

Total protein and cytosolic extracts were prepared by using modified RIPA buffer and Fermentas Proteojet Cytoplasmic and Nuclear Protein Extraction Kit (K0311), respectively. For immunoblotting equal volumes of total or cytosolic lysates were loaded onto an SDS polyacrylamide gel for electrophoretic analysis. The proteins in the gel were transferred onto PVDF membranes (Pierce), which were first blocked with Tris-buffered saline with 0.1% Tween-20 (TBST) containing 5% nonfat dry milk for 1 h at room temperature. The membrane then was blotted with primary antibodies against phospho-Met (Y-1234/1235) cell signaling 3129, MUC1 (VU4H5) sc-7313, MUC1 cell signaling 4538, phospho-p44/42 Erk1/2 (MAPK) (Thr202/Tyr204) cell signaling 9101, MAPK (ERK1) (C-16) sc-93, β-catenin (E-5) sc-7963, phospho-β-catenin cell signaling 9566S), vimentin BD-550513, c-Myc (sc-40) calnexin (sc-11397), cytokeratin-18 (sc-51582), lamin A/C (sc-7293) in TBST containing 3% NFDM, and Met (sc-161) in phosphate buffer saline containing 0.1% Tween-20 and 3% bovine serum albumin overnight at + 4°C. Proteins were detected by HRP-conjugated anti-rabbit (Pierce) and anti-mouse secondary antibodies (Pierce), with visualization by the ECL detection system (Pierce). The specific bands were recorded on X-ray film. Equal loading and transfer were confirmed by repeat probing for calnexin and Coomassie Blue Staining of proteins in gels. Band intensities were quantified as pixels by using ImageJ software (NIH). For quantitative determination of MUC1 antigen in conditioned media the Access Family of Immunoassay Systems (MUC1 (CA-15-3), IM2397 Beckman Coulter) was used. The MUC1 epitope (located within the 20-residue tandem repeat domain, SAPDTRPA) is recognized by B27.29 and DF3 monoclonal antibodies in this system.

### siRNA mediated knock down of MUC1

To silence MUC1, Mahlavu cells were transfected with 500nM siRNA (ON-TARGETplus SMARTpool siRNA L-004019-01-0020 Dharmacon) for 72 h using Fugene HD Transfection Reagent (Ref 04 709 705 001). Non-targeting siRNA (control siRNA-A: sc-37007) was used as scramble control. After 72 h incubation, cells were harvested or replated for subsequent experiments.

### Motility and invasion assay

*In-vitro* motility and invasion assays were performed as described [28]. The migration of Mahlavu cells was measured by Biocoat Cell Environment control inserts (8-μm pore size; BD Biosciences). Invasion assays with the same cell line were carried out using Matrigel Invasion Chambers (BD Bioscience). Briefly, cells transiently transfected with 500 nM MUC1 siRNA (as described above) or cells pretreated overnight with 1.5 μM SU11274 in DMEM with 5% FBS were placed in upper chambers. Untreated and control siRNA treated cells were used as controls. 5,000 cells were inoculated into each chamber. After 24 h incubation at 37^o^C, the medium was removed and cells were fixed and stained with Diff Quick (Siemens Healthcare Diagnostics). Cells on the upper portion of the membrane were wiped off with a cotton-tipped swab and cells that had traversed through the membrane were counted using a bright-field inverted microscope. Total cell numbers were counted for each chamber. Experiments were performed in at least triplicates. Bars represent fold differences in mean migrating or invading cell numbers. Fold differences were calculated by dividing the experimental results by the control results.

### Cell adhesion and proliferation assay

SiRNA or SU11274 pretreated and untreated and/or HGF stimulated Mahlavu cells were plated on the 96 E-Plate (Roche). Adhesion and proliferation were monitored in a real-time cell electronic sensing RT-CES system (xCeLLigence-Roche Applied Science) for 96 h. These experiments were performed in at least triplicate.

### Histopathology

Tissue samples were obtained from 42 patients with HCC and a cirrhotic history and 26 patients with only cirrhosis. They all had received transplants in Dokuz Eylul University, Izmir, Turkey. Normal donor liver biopsies were used as controls. The study was approved by the Ethics Committee of Dokuz Eylul University Medical School. Written informed consents were obtained from patients before liver transplantation or liver biopsy sampling. All tissue samples were fixed in formalin and embedded in paraffin. Archival materials of the patients were reevaluated by a certified pathologist (ÖS) for the confirmation of the diagnosis and to choose the most appropriate tissue block for immunohistochemistry. The histopathological analyzes of all patients were carried out by the WHO histopathological classification of liver and intrahepatic bile ducts [29]. Standard 5 μm tissue sections were taken on lysine-coated slides.

### Immunohistochemical procedure

Sections were deparaffinized in xylene and then rehydrated. Immunostaining was performed using an automated immunohistochemical stainer according to the manufacturer’s guidelines (Biogen, Lab vision autostainer 360). The antigen retrieval was performed by treatment of proteinase K for 20 min at 37^o^C. Endogenous peroxidase activity was blocked by incubation with 3% H_2_O_2_ for 15 min at room temperature. Tissues were incubated for 9 min with avidin-biotin blocking solution (SkyTek Lab.), and then primary antibodies anti-MUC1 (sc-7313) or anti-c-Met (sc-161) were applied at 1:100 dilutions and incubated for 35 min. The sections were stained with 3, 3-diaminobenzidine tetrahydrocloride (DAB), a chromogen stain (brown in color), and counterstained with hematoxylin.

### Evaluation of staining

All staining was semi-quantitatively evaluated by a certified pathologist (ÖS). Expression of c-Met was defined as membranous and/or cytoplasmic when more than 10% of the hepatocytes stained positive for c-Met. The extent of staining was scored as one positive (10% to 25% of cells were positively stained), two positive (25%-50% of cells were positively stained) and three positive (more than 50% of the cells were positively stained). Staining with MUC1 antibody was defined as cytoplasmic and canalicular staining in hepatocytes and the extent of staining was again scored semi-quantitatively. If less than 10% of hepatocytes expressed MUC1 antibody this staining was scored as one positive staining between 10% and 30% of cells, and staining in more than 30% of cells were regarded as two and three positive, respectively. The intensity of MUC1 and c-Met immunostaining was semiquantitatively graded as follows: none (0), weak (+1), moderate (+2), and intense (+3).

### Statistical analysis

All data for motility and invasion assays were expressed as mean ± S.E. Statistical analysis was performed using the GraphPad Prism and Statistical Package for Social Sciences 15.0 (SPSS Inc., Chicago, IL, USA). Statistical methods included Analysis of variance (ANOVA), Mann–Whitney U test, and *×*^2^-test. ANOVA was used in the case of comparison of multiple groups. Mann–Whitney U test and *×*^2^-test were used for the evaluation between two points as appropriate. Overall survivals of cells were computed using the Kaplan-Meier method and comparison between groups were analyzed using the log-rank test. Correlation between two groups was assessed by Pearson’s correlation analysis. p < 0.05 was considered statistically significant.

## Abbreviations

HCC: Hepatocellular carcinoma; HGF: Hepatocyte Growth Factor; IP: Immunoprecipitation; MUC1: Mucin1; siRNA: Small interfering RNA; SE: Standard Error.

## Competing interests

The authors declare that they have no competing interests.

## Authors’ contributions

GB and PK carried out western blot and co-immunoprecipitation experiments and drafted the manuscript with NA. PK and MC both participated in experimental procedures related with MUC1 silencing. PK also carried out motility and invasion experiments. ÖS performed the immunohistochemical analysis on tissue samples and analyzed them. SK provided tissue samples and clinical data. NA supervised project, made substantial contributions to conception and design of the study, analysis and interpretation of data, and wrote the main manuscript. EE gave technical support and conceptual advice and edited the manuscript for intellectual content. CK performed cell line authentication experiments and critically edited the manuscript. All authors read and approved the final version of manuscript.

## Supplementary Material

Additional file 1 Figure S1. Effect of HGF stimulation on c-Met expression in MUC1 negative and, MUC1 over-expressed HCC cells. MUC1 negative well-differentiated HuH-7 cells (A) and MUC1 over-expressing poorly-differentiated cells SNU-449 (B) and Mahlavu (C) were treated with HGF at the indicated times. After treatment, cells were lysed and subjected to immunoblotting using anti-c-Met and anti-calnexin antibodies. Calnexin was used as a loading control. Click here for file

Additional file 2 Figure S2. Investigation of MUC1 in total and cytosolic cell extracts and conditioned media. Overnight starved Mahlavu cells were treated with medium alone and with HGF for 15, 30, 60 min. Conditioned media were collected and total protein extracts obtained at the indicated time points after HGF administration. Then total protein extracts and conditioned media analyzed by Western Blotting for MUC1 expression (A). Simultaneously cytosolic cell extract were prepared and analyzed for MUC1 expression. Cytokeratin-18 used for equal loading and transfer control for cytosolic extracts. Lamin A/C used for verifying that nuclear protein did not leak into the cytosolic cell fraction during cell fractionation (B). Click here for file

Additional file 3 Table S1. P53 status and the expression levels of MUC1 and c-Met in HCC cell lines. (NF: non-functional, F: functional, ND: not determined, high: high level protein expression, low: low level protein expression). Click here for file
