# Supplementary figures and images for: Cooperative interaction of MUC1 with the HGF/c-Met pathway during hepatocarcinogenesis
Source: Mol Cancer. 2012 Sep 11;11:64. doi: 10.1186/1476-4598-11-64 (PMC3542123; doi:10.1186/1476-4598-11-64)

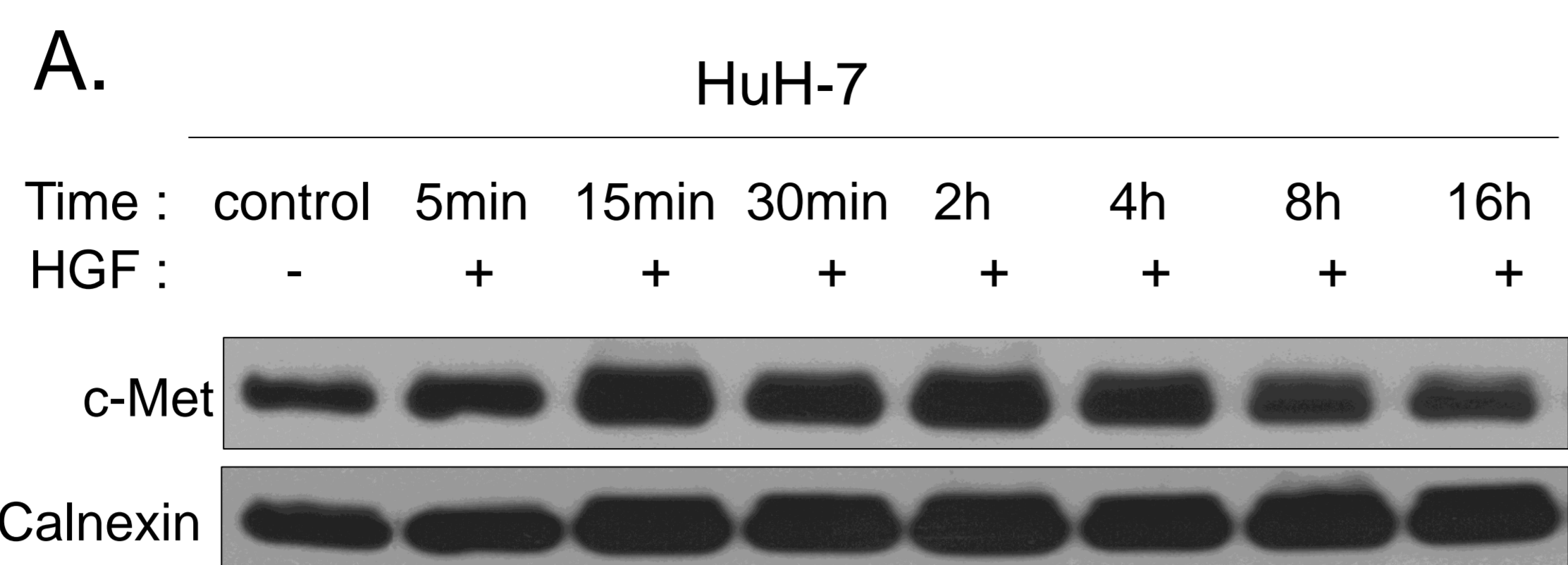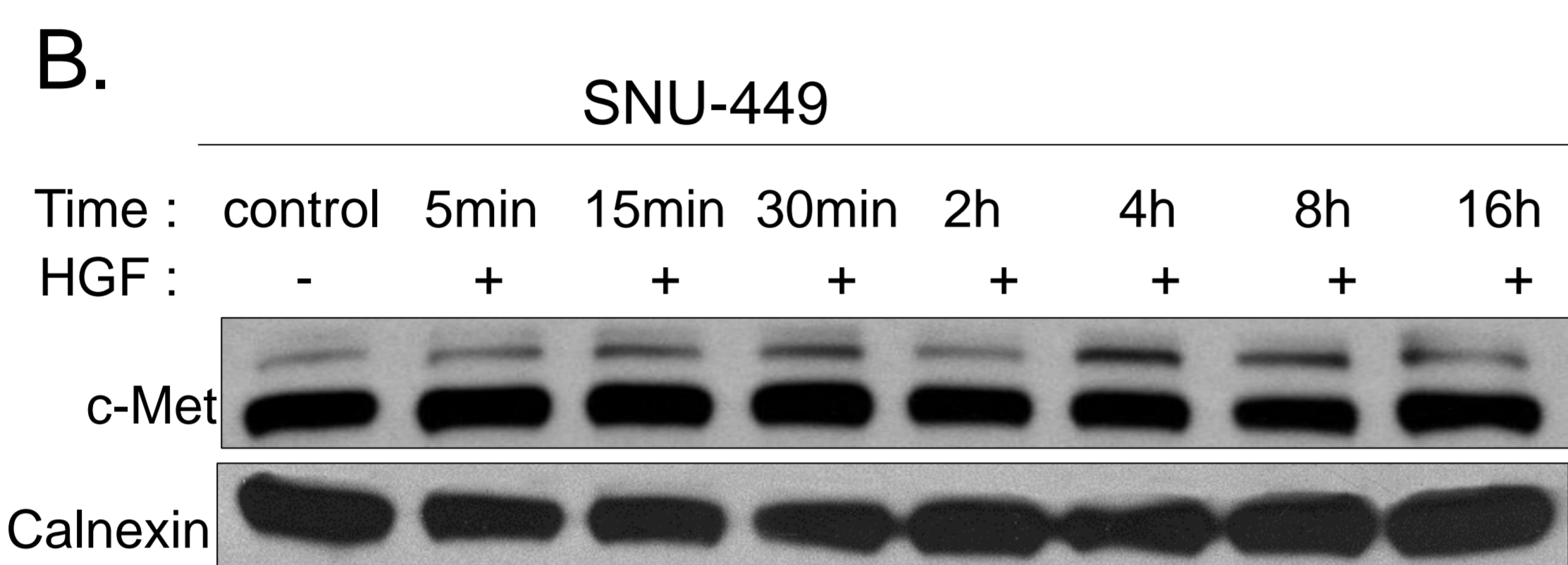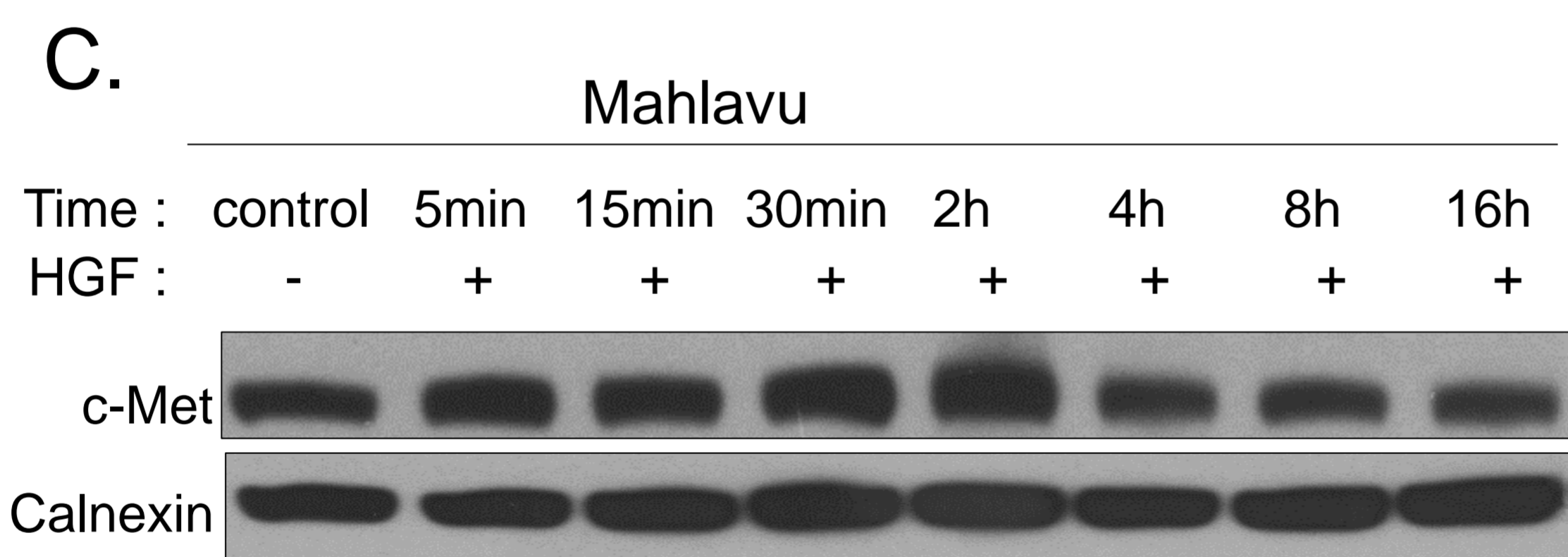

Supplementary Figure 1

Supplement: Additional file 1 — Figure S1. Effect of HGF stimulation on c-Met expression in MUC1 negative and, MUC1 over-expressed HCC cells. MUC1 negative well-differentiated HuH-7 cells (A) and MUC1 over-expressing poorly-differentiated cells SNU-449 (B) and Mahlavu (C) were treated with HGF at the indicated times. After treatment, cells were lysed and subjected to immunoblotting using anti-c-Met and anti-calnexin antibodies. Calnexin was used as a loading control. [file 1476-4598-11-64-S1.pdf]

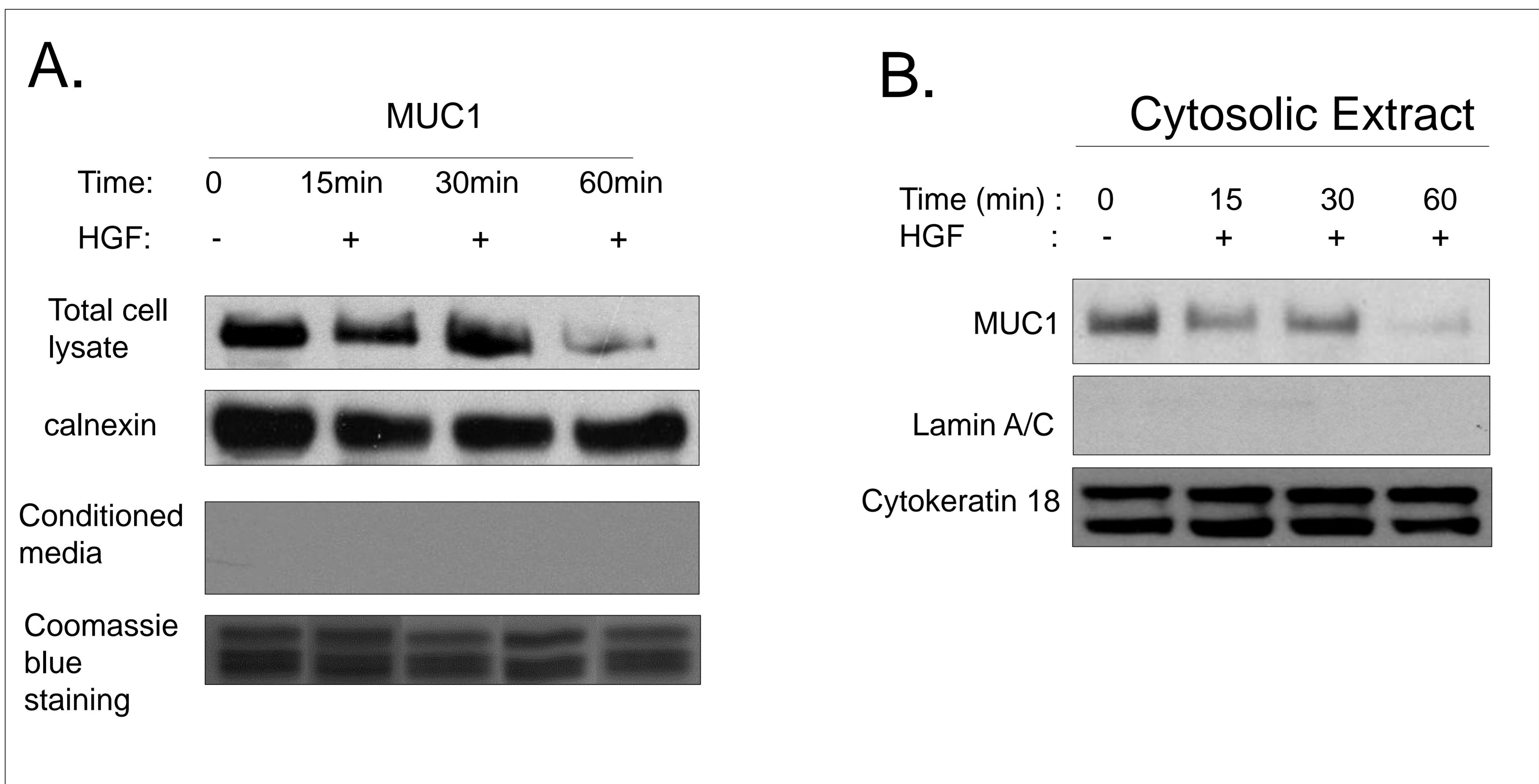

Supplementary Figure 2

Supplement: Additional file 2 — Figure S2. Investigation of MUC1 in total and cytosolic cell extracts and conditioned media. Overnight starved Mahlavu cells were treated with medium alone and with HGF for 15, 30, 60 min. Conditioned media were collected and total protein extracts obtained at the indicated time points after HGF administration. Then total protein extracts and conditioned media analyzed by Western Blotting for MUC1 expression (A). Simultaneously cytosolic cell extract were prepared and analyzed for MUC1 expression. Cytokeratin-18 used for equal loading and transfer control for cytosolic extracts. Lamin A/C used for verifying that nuclear protein did not leak into the cytosolic cell fraction during cell fractionation (B). [file 1476-4598-11-64-S2.pdf]
