# Supplementary material for: Cooperative interaction of MUC1 with the HGF/c-Met pathway during hepatocarcinogenesis
Source: Mol Cancer. 2012 Sep 11;11:64. doi: 10.1186/1476-4598-11-64 (PMC3542123; doi:10.1186/1476-4598-11-64)
Supplement: Additional file 3 — Table S1. P53 status and the expression levels of MUC1 and c-Met in HCC cell lines. (NF: non-functional, F: functional, ND: not determined, high: high level protein expression, low: low level protein expression). [file 1476-4598-11-64-S3.doc]

**Additional file** 3 **Table S1:** P53 status and the expression levels of MUC1 and c-Met in HCC cell lines. (NF: non-functional, F: functional, ND: not determined, high: high level protein expression, low: low level protein expression)

| **Cell lines** | **p53 status** | **MUC1 expression** | **c-Met expression** |
| --- | --- | --- | --- |
| **HuH7** | NF | ND | low |
| **Hep 3B** | NF | ND | low |
| **Hep G2** | F | ND | low |
| **SNU-449** | NF | high | high |
| **SNU-475** | NF | high | high |
| **Mahlavu** | NF | high | high |
